# EEG source connectivity to localize the seizure onset zone in patients with drug resistant epilepsy

**DOI:** 10.1016/j.nicl.2017.09.011

**Published:** 2017-09-14

**Authors:** Willeke Staljanssens, Gregor Strobbe, Roel Van Holen, Vincent Keereman, Stefanie Gadeyne, Evelien Carrette, Alfred Meurs, Francesca Pittau, Shahan Momjian, Margitta Seeck, Paul Boon, Stefaan Vandenberghe, Serge Vulliemoz, Kristl Vonck, Pieter van Mierlo

**Affiliations:** a Medical Image and Signal Processing Group, Department of Electronics and Information Systems, Ghent University - imec, De Pintelaan 185, 9000 Ghent, Belgium; b Epilog, Vlasgaardstraat 52, 9000 Ghent, Belgium; c Laboratory for Clinical and Experimental Neurophysiology, Neurobiology and Neuropsychology, Department of Neurology, Ghent University Hospital, De Pintelaan 185, 9000 Ghent, Belgium; d EEG and Epilepsy Unit, Neurology Department, University Hospitals and Faculty of Medicine of Geneva, Rue Gabrielle-Perret-Gentil 4, 1205 Geneva, Switzerland; e Department of Neurosurgery, University Hospitals of Geneva and University of Geneva, rue Gabrielle-Perret-Gentil 4, 1205 Geneva, Switzerland; f Functional Brain Mapping Lab, Department of Fundamental Neurosciences, University of Geneva, rue Gabrielle-Perret-Gentil 4, 1205 Geneva, Switzerland

**Keywords:** Ictal imaging, EEG source imaging, Functional connectivity, Clinical EEG, Granger causality

## Abstract

Electrical source imaging (ESI) from interictal scalp EEG is increasingly validated and used as a valuable tool in the presurgical evaluation of epilepsy as a reflection of the irritative zone. ESI of ictal scalp EEG to localize the seizure onset zone (SOZ) remains challenging. We investigated the value of an approach for ictal imaging using ESI and functional connectivity analysis (FC). Ictal scalp EEG from 111 seizures in 27 patients who had Engel class I outcome at least 1 year following resective surgery was analyzed. For every seizure, an artifact-free epoch close to the seizure onset was selected and ESI using LORETA was applied. In addition, the reconstructed sources underwent FC using the spectrum-weighted Adaptive Directed Transfer Function. This resulted in the estimation of the SOZ in two ways: (i) the source with maximal power after ESI, (ii) the source with the strongest outgoing connections after combined ESI and FC. Next, we calculated the distance between the estimated SOZ and the border of the resected zone (RZ) for both approaches and called this the localization error ((i) LE_pow_ and (ii) LE_conn_ respectively). By comparing LE_pow_ and LE_conn_, we assessed the added value of FC. The source with maximal power after ESI was inside the RZ (LE_pow_ = 0 mm) in 31% of the seizures and estimated within 10 mm from the border of the RZ (LE_pow_ ≤ 10 mm) in 42%. Using ESI and FC, these numbers increased to 72% for LE_conn_ = 0 mm and 94% for LE_conn_ ≤ 10 mm. FC provided a significant added value to ESI alone (*p* < 0.001). ESI combined with subsequent FC is able to localize the SOZ in a non-invasive way with high accuracy. Therefore it could be a valuable tool in the presurgical evaluation of epilepsy.

## Highlights

• ESI + functional connectivity analysis allows localizing the SOZ with high accuracy.
• Functional connectivity analysis offered a significant added value to ESI.
• The method is robust for inter- and intra-patient variability.
• The method could be a useful tool in the presurgical evaluation of epilepsy.

## 1 Introduction

For epilepsy patients who do not respond to anti-epileptic drugs, surgery is an important treatment option (de Tisi et al., 2011, Wiebe et al., 2001). To obtain successful outcome following epilepsy surgery, the delineation of the epileptogenic zone (EZ) is necessary. Unfortunately, there is no individual method to localize this EZ. Epileptologists make a deliberate estimation based on the integration of the results gained through the presurgical evaluation protocol. Magnetic Resonance Imaging (MRI) and visual inspection of the interictal and ictal (video-) electroencephalogram (EEG) with the determination of the irritative zone (IZ) and seizure onset zone (SOZ), respectively, are cornerstone investigations in this protocol (Carrette et al., 2011). However, SOZ estimation based on the visual inspection of the video-EEG is labor-intensive, time-consuming, to a certain extent subjective, and sometimes impossible. In some cases, invasive EEG (iEEG) is required to localize the SOZ, which is associated with potential complications (Hamer et al., 2002, Lee et al., 2000). Therefore, it would be of high clinical value to have a method that is able to identify the SOZ in a non-invasive, objective, and fast way with high accuracy in order to better target or avoid iEEG.

During the last decades, increased computational power and advanced signal processing techniques enabled novel epilepsy research dedicated to this purpose. Methods based on non-invasive scalp EEG are preferred, not only because it is cheap, portable and safe, but also because it corresponds to the most unambiguous biomarker of epileptic activity. Moreover, non-invasive EEG is routinely performed during long-term presurgical investigations. Although EEG has a high temporal resolution in the order of milliseconds, its main challenge is the fact that the recorded potentials are subject to volume conduction. Neuronal activity is attenuated and distorted before it reaches the electrodes. EEG source imaging (ESI) can overcome the volume conductor problem as it estimates the underlying brain activity generated by the recorded potentials.

ESI of interictal activity is an established technique to determine the IZ. However, the IZ may designate a (partially) different, possibly larger region than the SOZ that needs to be resected. ESI of ictal activity is more challenging, due to the less frequent occurrence of seizures than interictal spikes and the many artifacts during seizures, but potentially more informative for surgery guidance as it reflects the SOZ directly (Elshoff et al., 2013, Jayakar et al., 1991, Rémi et al., 2011).

Previous studies have tried to localize the SOZ based on merely ESI of ictal EEG with varying results (Assaf and Ebersole, 1997, Boon et al., 2002, Ebersole, 2000, Habib et al., 2015, Jung et al., 2009, Koessler et al., 2010, Lantz et al., 1999, Pellegrino et al., 2016, Wang et al., 2011, Yang et al., 2011). Yet, within the concept of a network disorder, more sophisticated approaches may be required to correctly identify the SOZ from scalp EEG recordings. During a seizure, several brain regions participate in the epileptic network, and the challenge is to isolate the main driver(s) of the network from the secondarily activated regions, rather than finding the sources with the highest energy (Richardson, 2012, Spencer, 2002). Functional brain connectivity analysis (FC) can be used to analyze the relationship between the sources calculated by ESI (Van de Steen et al., 2016), and may thus provide a more reliable SOZ localization. In this paper, we adapt a previously published method by our group (Staljanssens et al., 2017) that combines ESI and FC based on Granger causality. Whereas in the previous study the focus was on high-density EEG (hd-EEG), the adapted version of the algorithm allows for successful analysis of clinical/low-density EEG. We validated the approach in the clinical scalp ictal EEG recording of 111 seizures from 27 patients (23 with temporal lobe epilepsy) who were rendered seizure-free following resective surgery.

## 2 Methods

### 2.1 Patients

27 patients, 18 from Ghent University Hospital and 9 from Geneva University Hospital, were included based on the following criteria: (1) drug-resistant epilepsy; (2) availability of EEG recordings of at least one seizure, recorded with at least 27 electrodes; (3) a single resective surgery procedure of the supposed epileptogenic zone; (4) surgical outcome Engel Class I with a minimal post-operative follow-up of 12 months; (5) availability of pre- and post-operative T1-weighted MRI. Table 1 lists the main patient characteristics. The local ethical committees approved the study and all patients gave written informed consent.

**Table 1:** Patient details. Patients 1–17 are from Ghent University Hospital and Patients 18–26 are from Geneva University Hospital. EMU = epilepsy monitoring unit, PAT = Patient, LE = lobe epilepsy, R = right, L = left, T = temporal, F = frontal, P = Parietal, O = occipital, C = central, inf = inferior, ant = anterior HEM = hemisphere, HIP = hippocampal, DNET = dysembryoplastic neuroepithelial tumor, IED = interictal epileptiform discharges, CPS = complex partial seizure, SPS = simple partial seizure, AH = amygdalohippocampectomy, S = selective, y = year, m = month.

PAT nr.	Epilepsy type	Age at epilepsy at onset (y)	Age at surgery (y)	EMU interictal EEG	EMU ictal EEG	Invasive EEG	MRI	Surgery	Follow-up duration (m)
1	RFLE	8	30	Bursts of bilateral slow sharp waves during sleep	Absence of clear ictal discharges, some bilateral and/or RF sharp activity	Low voltage fast activity on electrode grid contacts above cortical dysplasia	RF opercular focal cortical dysplasia	RF opercular lesionectomy	48
2	RTLE	5	48	RFT IED	RFT rhythmic delta activity	/	R HIP atrophy	R SAH	36
3	RTLE	6	33	RFT IED	RT and hemispheric rhythmic theta activity	/	R HIP atrophy and secondary sclerosis of R ant T pole	R SAH	36
4	RTLE	15	22	RFT IED	RFT rhythmic theta-delta activity	/	R HIP atrophy	R SAH	36
5	RTLE	18	55	RFT IED	RFT and CP rhythmic theta activity	Low voltage fast activity RT neocortical grid electrodes + early R HIP electrode involvement	No epileptogenic lesion	RT neocortical topectomy + SAH	33
6	RTLE	20	35	RFT IED	RFT rhythmic theta-delta activity with early L HEM involvement	/	R gyrus T inf DNET	R basoT lesionectomy	18
7	RTLE	12	27	RFT IED	RFT rhythmic theta activity	/	R HIP atrophy	R SAH	29
8	LOLE	48	49	LF & LFT IED	LPO low voltage fast activity & LT rhythmic theta activity	Low voltage fast activity on LO grid electrode contacts	LO cystic lesion	LO lesionectomy	25
9	LTLE	19	54	LF & LT IED & infrequent RT IED	L generalized decrement followed by LT rhythmic theta activity	L HIP fast & rhythmic polyspike activity	L HIP atrophy	L SAH	21
10	LTLE	18	28	LFT IED	L HEM or bilateral rhythmic theta-delta activity	L HIP rhythmic spike activity	LT cavernous hemangioma	L 2/3 ant T lobectomy	20
11	RTLE	24	50	RFT IED	RFT rhythmic theta activity	/	R HIP atrophy	R SAH	12
12	RTLE	24	26	RFT IED	Bilateral T rhythmic theta activity	R HIP and inf T rhythmic activity	R ant inf T gyrus abnormal structure	R 2/3 ant T lobectomy	65
13	LTLE	31	36	LFT IED	LFT rhythmic activity	/	Lesion L inf T gyrus	LT lesionectomy	56
14	LTLE	40	49	LFT IED	LFT rhythmic theta-activity	Rhythmic low voltage delta activity with spiking on basal ant T grid electrodes with early spread to HIP electrodes	LT, L precentral & inf P posttraumatic atrophy	L 2/3 ant T lobectomy + corticectomy of post basal T neocortex	47
15	RTLE	35	42	RFT IED	RFT rhythmic delta activity	/	R HIP atrophy	R SAH	26
16	LTLE	36	40	No IED	LFT rhythmic delta activity	/	L HIP atrophy	L SAH	48
17	RTLE	4	18	RFT IED	R HEM theta-delta activity	/	R HIP atrophy	R SAH	48
18	LTLE	31	43	LFT slow sharp waves	L HEM rhythmic delta activity	/	L HIP atrophy	L SAH	12
19	RTLE	11	16	RT IED	RT delayed onset with slow sharp waves	R amygdala/HIP rhythmic beta activity	R HIP atrophy	R 2/3 ant T lobectomy	60
20	LFLE	1	12	LCP IED	LFC beta rhythm evolving to FC rhythmic sharp waves	LF tuber rhythmic beta activity evolving to LFC tuber rhythmic beta activity	Tuberous sclerosis	LF and LFC lesionectomy (tuber)	60
21	LTLE	1	11	Bilateral FT IED & LT slow waves	L HEM rhythmic slowing propagating to R HEM, then LT rhythmic theta activity	L lateral temporal	No epileptogenic lesion	L 2/3 ant T lobectomy sparing AH	96
22	LTLE	1	12	Multifocal L IED	Successive bursts of rhythmic polyspike-waves with LFC onset	RT pole rhythmic beta activity propagating to basal RT	Tuberous sclerosis	L 2/3 ant T lobectomy	48
23	RTLE	9	32	RT IED	LPO rhythmic theta activity	R HIP rhythmic beta activity evolving to alpha and delta	R HIP atrophy	R 2/3 ant T lobectomy	84
24	RTLE	25	37	RFT IED	RT rhythmic theta activity ➔ RT rhythmic spiking ➔ contralateral diffusion	/	R HIP atrophy	R 2/3 ant T lobectomy	54
25	RTLE	20	43	RTP IED	RT rhythmic theta activity	/	R HIP atrophy	R 2/3 ant T lobectomy	48
26	RTLE	20	37	RTP IED	Rhythmic RFT delta activity with spikes ➔ R HEM sharp waves max. FT	/	RF focal cortical dysplasia	RF lesionectomy	48
27	RTLE	25	30	RT IED	R HEM rhythmic alpha activity with max RT	/	RT pole and amygdala dysplasia	R 2/3 ant T lobectomy with limited HIP resection	24
Mean		18.8	33.9						42.1
Median		19	35						47
Std		12.6	13.1						20.5

### 2.2 EEG recording

The patients of Ghent University Hospital (PAT1 – PAT18) underwent long-term video EEG monitoring (Micromed, Treviso, Italy) lasting 3–8 days. A setup with 27 electrodes was used of which 21 were placed according to the International 10–20 system. Additionally, 3 electrodes were placed in zygomatic, preauricular, and mastoid regions on both sides of the head (F9–F10, T9–T10 and Tp9–Tp10 respectively) (Boon et al., 1997). The sampling frequency was 256 Hz.

For the 9 patients of Geneva University Hospital (PAT19 – PAT27), ictal EEG recordings, lasting at least 24 h, with 29–32 electrodes placed according to the international 10–10 system were available with a sampling rate of either 250 Hz or 256 Hz.

### 2.3 EEG preprocessing and ictal epoch selection

EEG preprocessing was done in BrainVision Analyzer (BrainProducts GmbH, Germany). The patient-data was band-pass filtered between 1 and 30 Hz to remove baseline drift and to reduce high-frequency muscle artifacts. An extra notch filter at 50 Hz was applied to filter out remaining power line noise. For all recorded seizures, together with an experienced epileptologist (KV, SV), a (quasi) artifact-free epoch close to the electrographic onset that was representative for the seizure was selected. If no clear EEG changes were observed, the clinical onset was used instead. The epochs were as long as possible, with a minimum of 1 s and a maximum of 5 s. Additional preprocessing was performed to increase signal-to-noise ratio: when bad quality channels were present, they were spatially interpolated using splines instead of removed, allowing to use the same analysis pipeline for every epoch of each patient. In case of long lasting muscle artifact, an extra band-pass filter between 1 and 10 Hz was applied. For eye blink or cardiac artifact removal, ICA was used (Makeig et al., 1996) using the restricted fast ICA (Hyvarinen et al., 2001) implementation in BrainVision Analyzer on the available EEG channels. Only components showing exclusively clear artifactual activity, namely eye blinks with clear frontal topographic pattern (in 25 seizures) and cardiac artifact (in 1 seizure), were removed. The selected epochs were common average referenced and their fundamental seizure frequency band (Frequency band of interest, FOI) was determined as the band with maximal global field power using the Fast Fourier Transform (FFT).

### 2.4 From ictal epoch to SOZ

We used two methods to localize the SOZ from the selected ictal EEG epoch. The first method was based solely on ESI, and named “ESI power”. The second method was based on ESI with subsequent FC and named “ESI + connectivity”. Both methods have been extensively described in previous research (Staljanssens et al., 2017). The main difference with this research is the segment selection (described above) and the fact that we calculate the power and connectivity values in a FOI rather than in the broadband spectrum. We summarize the methods below and highlight the differences.

After the selection and preprocessing of an ictal epoch as described in Section 2.3, ESI was applied. For this purpose, realistic finite difference method (FDM) head models consisting of six different tissues (air (0 S/m), scalp (0.33 S/m), skull (0.0132 S/m), cerebrospinal fluid (1.79 S/m), grey matter (0.33 S/m) and white matter (0.14 S/m)) were constructed based on the individual patient's pre-operative T1-weighted MR image (Montes-Restrepo et al., 2016, Strobbe et al., 2016). The solution space was constructed as a uniform grid in the segmented grey matter, excluding the cerebellum, with a spacing of 4 mm. An in-house implementation of LORETA was used as inverse solution method (Pascual-Marqui et al., 1994). LORETA solutions are typically smooth throughout the brain in which some hotspots of higher activity are apparent and that might partially overlap. We selected K hotspots as nodes or sources as possible SOZs for the subsequent analyses with the following approach. We considered the power distribution in the solution space over the complete duration of the analyzed epoch. For every solution point, we calculated the sphere power as the mean power of all solution points in a sphere centered on the considered solution point. Those solution points that had no neighbors with a higher sphere power than their own corresponded to local maxima in power and were selected as possible sources for the SOZ. By varying the radius of the sphere, more or less sources can be selected. In one extreme case, the radius is smaller than the grid resolution (here 4 mm), and all sources will be selected. In the other extreme case, the radius is larger than the largest distance between two solution points, and only one source will be selected, corresponding to the global maximum in power. In this case, subsequent connectivity analysis is impossible, since there is only one source. In the former case, subsequent network analysis might be biased since in a LORETA solution neighboring sources are correlated and thus spurious connections might be introduced. Therefore, choosing this radius is a trade-off between not making the search area unnecessarily large and not excluding possible epileptic network nodes. For a radius of 15 mm, we found for all seizures an acceptable amount of network nodes, which ranged between K = 4 and K = 24. Increasing the radius resulted in the undesirable situation that for some seizures only one network node was found and decreasing the radius increased the upper limit of the number of sources, making the search area unnecessarily large. To continue the analysis, we considered the time series of the selected sources. No constraints were applied on the orientation of these sources; so every selected source was represented by three time series, one for each orthogonal spatial dimension. We used Singular Value Decomposition (SVD) to represent every selected source by only one time series, namely the time series associated with the largest singular value of the SVD (Golub and Reinsch, 1970). For the ESI power method, we selected the source with maximal power in the FOI as the estimated SOZ.

For the ESI + connectivity method, FC based on Granger causality was applied on the time series of the selected sources to reveal the driver of the epileptic network. To this end, the data was modeled by a time-varying multivariate autoregressive (TVAR) model, of which the coefficients were estimated using the Kalman filtering algorithm (Arnold et al., 1998, Schlögl et al., 2000), with a model order of 10, an update coefficient of 10^− 4^ and a smoothing factor of 100, based on previous research (Astolfi et al., 2008, Coito et al., 2015, Staljanssens et al., 2017, van Mierlo et al., 2011, van Mierlo et al., 2013).

From the time-varying transfer matrix ***H***(*f*, *t*) of the model, the spectrum-weighted Adaptive Directed Transfer Function (swADTF) (van Mierlo et al., 2013) was calculated at every time sample *t* of the selected epoch of length *T* and for the FOI = [*f*_1_ *f*_2_] with a resolution of 0.1 Hz, as a measure for the information flow between every two of the *K*selected sources:

$$\text{swADT}F_{\mathrm{ij}}\left(t\right)=\frac{\sum_{f=f_{1}}^{f_{2}}{\left|H_{\mathrm{ij}}\left(f,t\right)\right|}^{2}\sum_{k=1}^{K}{\left|H_{\mathrm{jk}}\left(f,t\right)\right|}^{2}}{\sum_{l=1}^{K}\sum_{f\prime =f_{1}}^{f_{2}}{\left|H_{\mathrm{il}}\left(f^{\prime },t\right)\right|}^{2}\sum_{s=1}^{K}{\left|H_{\mathrm{ls}}\left(f^{\prime },t\right)\right|}^{2}}$$

Finally, the outdegree of every source *j* was calculated as the sum of the swADTF values to every other source:

$$OD_{j}=\sum_{k=1}^{K}\sum_{t=1}^{T}\text{swADT}F_{\mathrm{kj}}\left(t\right)$$

in which we defined *swADTF*_*jj*_ = 0. ESI + connectivity selected the source with the highest outdegree as presumed SOZ.

### 2.5 Validation

#### 2.5.1 Localization errors

Since all patients included in this study were seizure-free for at least one year after surgery, we assume that the SOZ was located inside the resected tissue. The final result for each of the methods was one source, i.e. one point in the grey matter of the patient. Therefore, we defined the localization error of both methods, ESI power and ESI + connectivity, as the distance between the border of the resected zone (RZ), segmented from the post-operative MRI, and the SOZ estimated by the corresponding method. The localization errors were named LE_pow_ and LE_conn_, respectively. If the selected source was inside the RZ, the LE was set to zero.

#### 2.5.2 Seizure level

We calculated LE_conn_ and LE_pow_ for every seizure, and determined the amount of seizures with LE = 0 mm and LE ≤ 10 mm for both methods, to account for the spatial resolution of ESI (cm-range), and the brain shift that can occur after resective surgery.

#### 2.5.3 Patient level

For every patient, we calculated the percentage of seizures that were estimated inside the RZ and within 10 mm of the border of the RZ.

Furthermore, the percentage of patients for who all of their seizures were localized within the given limits (0 mm and 10 mm) was determined.

#### 2.5.4 Intra-patient robustness

When at least two analyzed seizures from one patient were available, the robustness of both methods against intra-patient variability could be assessed. For every seizure, a final source was selected as the estimated SOZ. In the ideal case, we would find a source inside the RZ for all the seizures of a specific patient. In reality, different sources, both within and outside (i.e. the algorithm performs wrong, or there are multiple foci) the RZ, can be found.

We quantified the intra-patient spatial dispersion of patient *P* by calculating the geometrical centroid and standard distance SD to this centroid of the finally selected sources:

$$\mathrm{SD}\left(P\right)=\sqrt{\frac{\sum_{i=1}^{N}{\left(x_{i}-\mu_{x}\right)}^{2}+\sum_{i=1}^{N}{\left(y_{i}-\mu_{y}\right)}^{2}+\sum_{i=1}^{N}{\left(z_{i}-\mu_{z}\right)}^{2}}{N}}$$

in which *N* is the number of seizures for patient *P*, (*x*_*i*_, *y*_*i*_, *z*_*i*_) are the Cartesian coordinates of the estimated SOZ (finally selected source) for seizure *i* and (*μ*_*x*_, *μ*_*y*_*μ*_*z*_) are the Cartesian coordinates of the geometrical centroid, based on all *N* seizures for that patient *P*. When the standard distance remains low, the method is robust and the spatial dispersion could be informative for the epileptologist to find the SOZ. In contrast, a large standard distance (e.g. 8 cm) within a single patient may be a marker for a less reliable result.

#### 2.5.5 Subgroup analysis based on resected volume

We calculated the resected volume in every patient using the convex hull of the segmented RZ and divided the patient population into a small RZ subgroup and a large RZ subgroup based on the resected volume. We determined whether there was a significant difference in LE_pow_ and LE_conn_ between the two subgroups. To get a more complete insight in the influence of the resected volume, we repeated this subgroup analysis based on the distance to the center of the RZ.

#### 2.5.6 Statistical testing

In each of the aforementioned validation steps, the results for ESI + connectivity and ESI power were statistically compared using a Wilcoxon sign-rank test for non-normally distributed data. Statistical analysis between subgroups was done with Wilcoxon rank-sum tests for independent samples. All significant *p*-values (*p* < 0.05) were reported.

## 3 Results

### 3.1 Seizure level

**Fig. 1:**
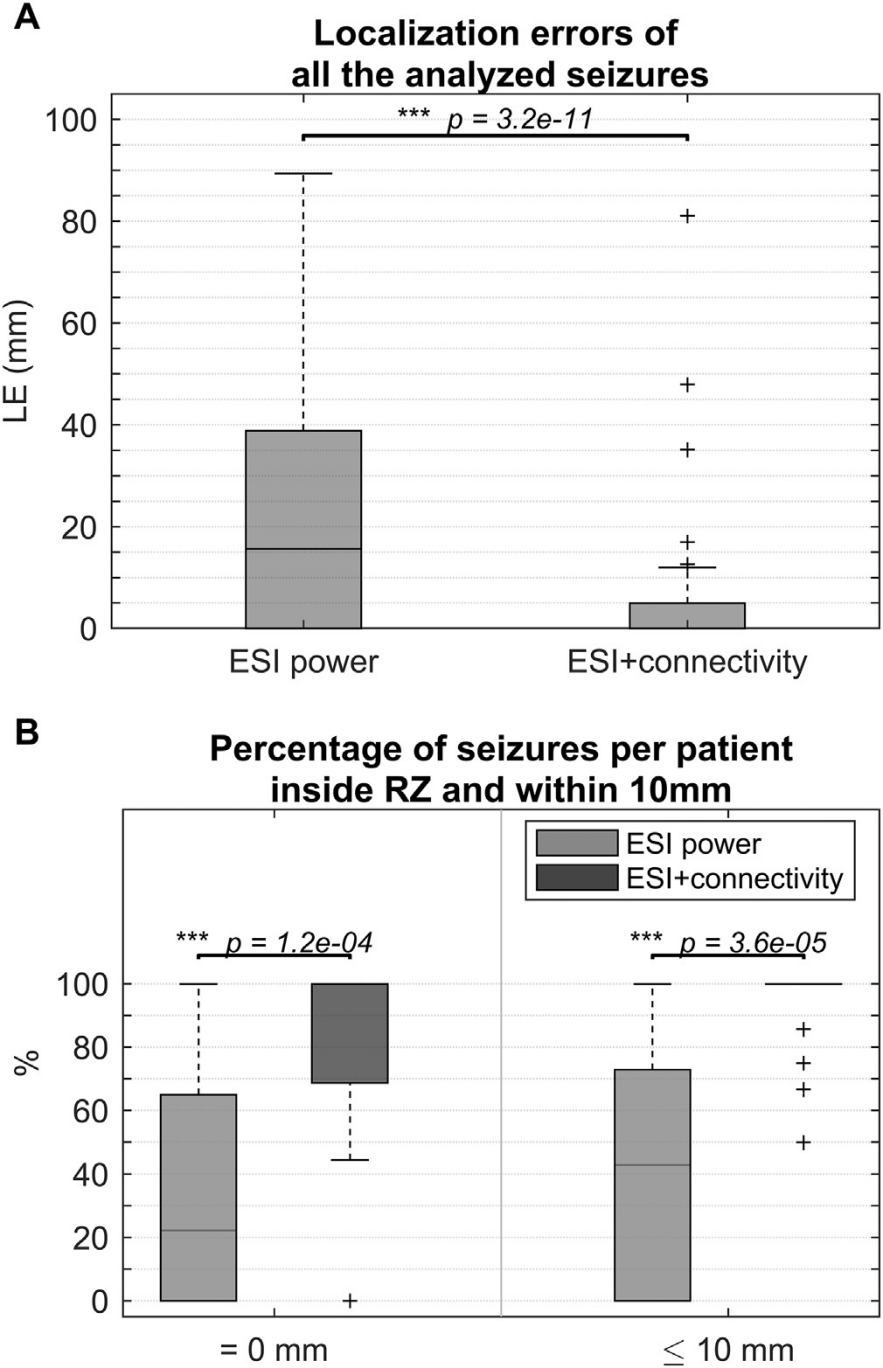
A) Boxplot of the localization errors of all analyzed seizures, B) percentage of correct localized seizures per patient, for both methods and both limits (LE = 0 mm and LE ≤ 10 mm).

In total, 111 seizures from 27 patients were analyzed (4.1 ± 2.9 seizures per patient). Table A.1 lists the selected epochs, the used frequency band of interest (FOI) and the applied preprocessing. Figs. A.1–A.3 of the Supplementary material show some examples of selected epochs. The localization errors for both the ESI power and the ESI + connectivity approach are shown for every patient and every seizure in Table 2. ESI power was able to localize the SOZ inside the RZ in 30.6% (34/111) of the seizures and within 10 mm of the border of the RZ in 42.3% (47/111) of the seizures. ESI + connectivity was inside or within 10 mm of the RZ in 72.1% (80/111) or 93.7% (104/111) of the seizures, respectively. The distribution of all the localization errors for ESI power and ESI + connectivity are shown in Fig. 1.A. The median localization error for ESI power was 15.7 mm in a range of 0–89.4 mm, for ESI + connectivity this was 0 mm in 0–81.1 mm. The distance to the border of the RZ was significantly lower for ESI + connectivity than for ESI power (*p* = 3.2 × 10^− 11^).

**Table 2:**
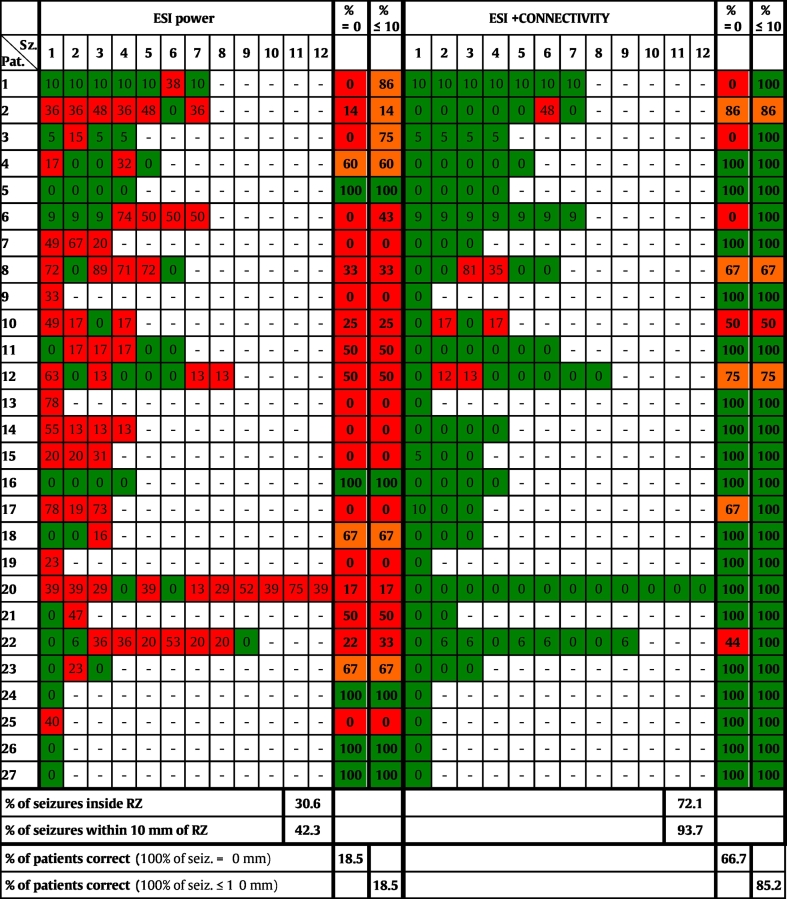
Overview of the localization errors of all analyzed seizures. Errors smaller than 10 mm (0 mm ≤ LE ≤ 10 mm) are colored green, errors larger than 10 mm (LE > 10 mm) are depicted in red. The percentage of seizures per patient localized inside and within 10 mm of the RZ is indicated. Percentages ≤ 50% are shown in red, between 50% and 100% are shown in orange and percentages equal to 100% are colored green. Pat. = Patient number, Sz. = number of analyzed seizure, RZ = border of resected zone.

### 3.2 Patient level

The percentage of seizures per patient that was estimated inside the RZ or within 10 mm of the border of the RZ, is shown in Table 2. For both limits, ESI + connectivity scored significantly better than ESI power (*p* = 1.2 × 10^− 4^ for LE = 0 mm and *p* = 3.6 × 10^− 5^ for LE ≤ 10 mm). This can be seen in Fig. 1.B.

ESI power was able to localize all seizures inside the RZ in only 18.5% (5/27) of the patients. This number stayed the same for seizures within 10 mm of the RZ. ESI + connectivity localized all seizures inside the RZ in 66.7% (18/27) of the patients and within 10 mm of the RZ in 85.2% (23/27) of the patients (see also Table 2).

### 3.3 Intra-patient robustness

**Fig. 2:**
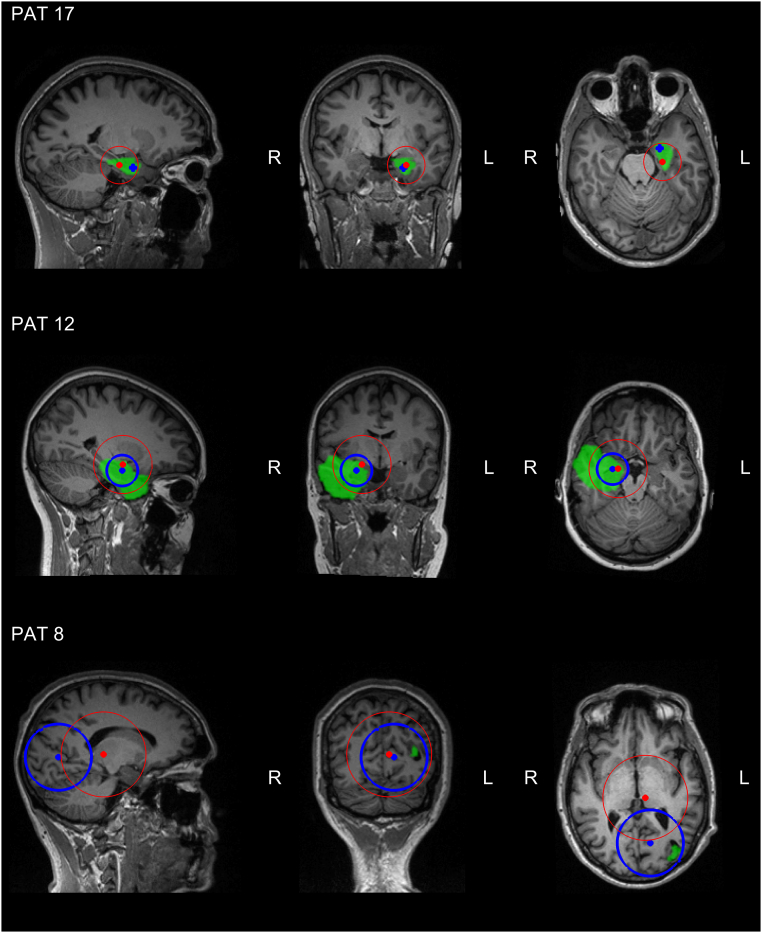
Three examples of the spatial dispersion of the estimated SOZs for ESI + connectivity (blue circle) and ESI power (red circle). The dot represents the centroid, whereas the circle represents the standard distance. The resected zone is highlighted in green. Both ESI + connectivity and ESI power gave a good indication of the SOZ in PAT 17, respectively 100% and 67% of the seizures were localized correctly. The spatial dispersion of ESI + connectivity points directly to the RZ with a standard distance equal to zero. However, the spatial dispersion of ESI power also gives a good indication where to look for the true SOZ, but less precise. In PAT 12, the standard distance for ESI + connectivity larger than zero, but the spatial dispersion is still informative, remaining mainly in the temporal lobe. The spatial dispersion based on ESI power, however, crosses lobe and even hemisphere borders and could be more difficult to interpret. For PAT 8, the spatial dispersion based on ESI + connectivity contains the RZ, whereas the spatial dispersion based on ESI power does not. Although, ESI + connectivity correctly localized 67% of the seizures, the standard distance is very high due to two completely wrong localizations, rendering the spatial dispersion less informative. (For interpretation of the references to color in this figure legend, the reader is referred to the web version of this article.)

**Fig. 3:**
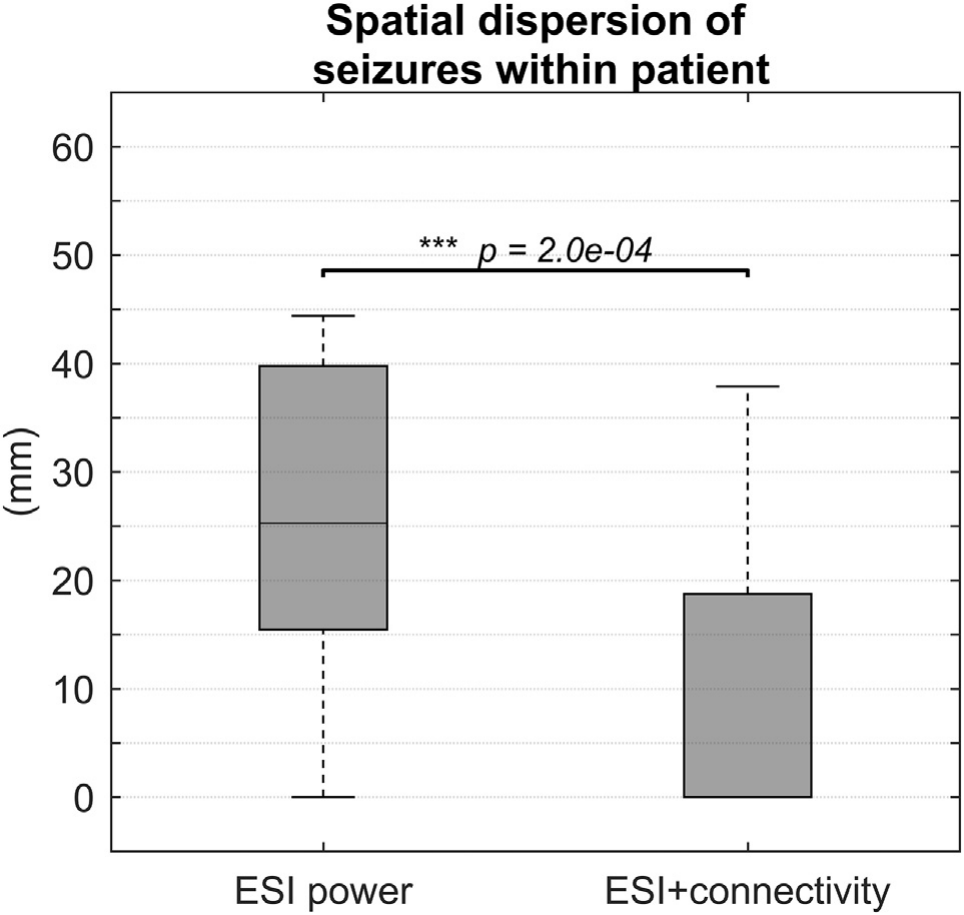
Distribution of the standard distance to the geometrical centroid within the patients who had more than one seizure during recording.

20 out of 27 patients had more than one seizure during recording (they had 5.2 ± 2.5 seizures on average). In Fig. 2, we depict the spatial dispersion obtained with both methods for three illustrative cases (2 TLE, 1 FLE) by a dot on the geometrical centroid and a circle with radius equal to the standard distance, centered at the centroid. This is overlaid on the pre-operative MRI of the patient, in which we highlight the ultimately resected zone in green. In Supplementary material A.2, the figures for all patients can be found. Fig. 3 shows a boxplot of all standard distances for both methods. ESI power had a median standard distance of 25.3 mm in a range of 0–44.4 mm, for ESI + connectivity this was 0 mm in a range of 0–37.9 mm. ESI power had a significantly higher standard distance than ESI + connectivity (*p* = 2.0 × 10^− 4^).

### 3.4 Subgroup analysis based on resected volume

**Fig. 4:**
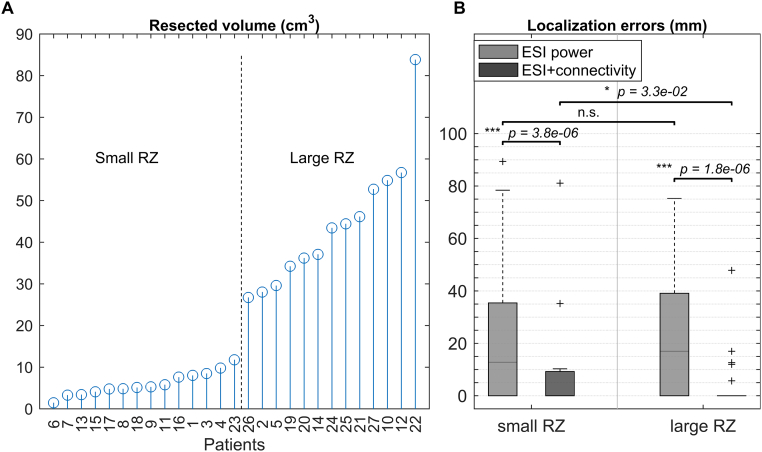
A) The resected volumes for every patient, sorted from small to large and B) boxplot of the localization errors corresponding to small and large resected volumes, for both methods.

The volumes and division into a small and large RZ subgroup can be seen in Fig. 4.A. Both for small and for large resections, we found that ESI + connectivity scored significantly better than ESI power (resp. *p* = 3.8 × 10^− 6^ and *p* = 1.8 × 10^− 6^), shown in Fig. 4.B.

**Fig. 5:**
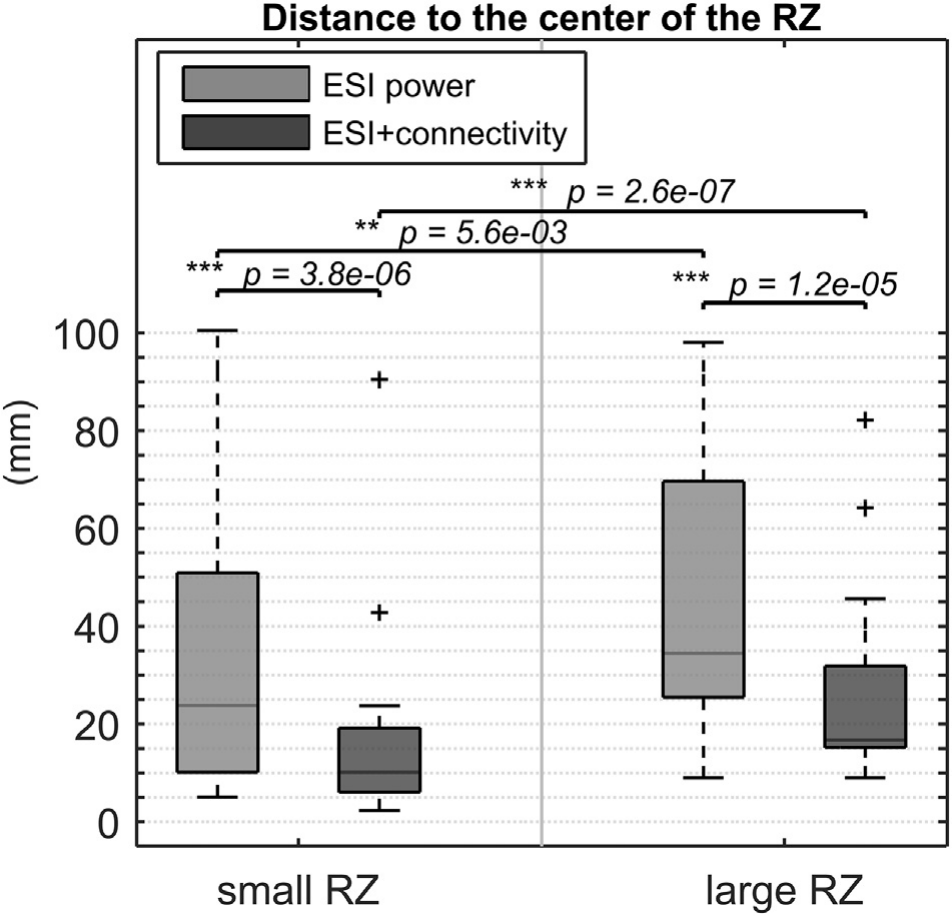
Distance to the center of the RZ for small and large resected volumes, for both methods.

ESI power for small resections had a median localization error of 12.81 mm in a range of 0–89.4 mm. For large resections, this was 17 mm in a range of 0–75.3 mm. There was no significant difference between small and large resections. For ESI + connectivity, we found a median localization error of 0 mm for both small and large resections. The range for small resections was 0–81.1 mm and for large resections 0–47.9 mm. A small significant difference was found in favor of large resections (*p* = 3.3 × 10^− 2^). Yet, there was no significant difference for small and large resections in the percentage of correctly localized seizures per patient. Moreover, when we repeated the analysis with the distance to the center of the RZ instead of the distance to the border of the RZ, we found significantly smaller values for small resections, for both methods, also shown in Fig. 5.

## 4 Discussion

### 4.1 Performance

In this paper, we found that ESI with subsequent FC is superior to localize the SOZ from non-invasive ictal EEG recorded using a clinical setup (≤ 32 electrodes), compared to ESI alone. We were able to indicate the SOZ inside the RZ and within 10 mm of the border of the RZ in 72.1% and 93.7% of the seizures, respectively. In 66.7% of the patients, all seizures were localized inside the RZ, and in 85.2% of the patients, all seizures were localized within 10 mm of the RZ. In contrast, ESI power was concordant in only 30.6% (0 mm error) or 42.3% (10 mm error) of the seizures. In 18.5% of the patients, all seizures were localized inside the RZ. This number stayed the same for the 10 mm tolerance. We were able to show that, in this framework, FC has a significant added value compared to ESI alone. The results of ESI power were comparable to Pellegrino et al. (2016), where for ESI a median localization error of 11 mm in a range of 0–87 mm was found, specifically when looking at small resections.

The superiority of ESI + connectivity over ESI power can be explained by two reasons. First, ESI power summarizes the time series of the selected network nodes into a single value, whereas ESI + connectivity investigates the time dynamics of the source signals. As a consequence, ESI + connectivity is probably better suited to deal with remaining artifacts and noise than ESI power. Second, ESI + connectivity allows localizing a driver that is more silent (less power) than the regions it influences.

Early SOZ localization studies mainly showed the feasibility of ictal ESI (Assaf and Ebersole, 1997, Jung et al., 2009, Lantz et al., 1999) and indicated the potential added value in the presurgical evaluation of epilepsy (Boon et al., 2002). However, in these studies, validation was performed in a more qualitative way, by assessing congruency between epilepsy diagnosis/surgery and localized SOZ on the lobar or sublobar level. During the last decade, with the emergence of more powerful forward modeling and inverse solution techniques, the quality and resolution of the solutions has increased to a degree that more quantitative, rigorous validation of ictal imaging methods is possible in terms of distance, correlation, spatial dispersion etc. To our knowledge, the first study reporting quantitative measures of SOZ localization quality dates from 2007 (Ding et al., 2007), reporting a localization error smaller than 15 mm in 85% of the seizures undergoing 31 electrode EEG recordings. We found a localization error smaller than 10 mm in > 90% of the patients. Other SOZ imaging studies reporting quantitative results made use of setups with at least 38 electrodes (Elshoff et al., 2013, Lu et al., 2012, Pellegrino et al., 2016, Staljanssens et al., 2017, Yang et al., 2011). Several methodological differences between these studies can be found, such as the used forward model and inverse solution for ESI, the possible application of FC and the FC measures, the frequency band of interest, etc. Although all of them show promising results, Lu et al. (2012) and Staljanssens et al. (2017) reported a drop in performance when fewer electrodes are used. We were able to increase the performance when using a lower density EEG setup by manually selecting and preprocessing adequate EEG epochs and limiting the analysis to the frequency band of interest of the seizure. However clinically highly recommended, most clinics unfortunately still lack the equipment to do (long-term) EEG monitoring with > 32 electrodes. This study is, to our knowledge, the first to achieve good performance based on low-density recordings, quantitatively validated in a more extensive patient population group than those that were used before, paving the way for a clinical use of the technique.

### 4.2 Intra-patient robustness

The standard distance to the centroid of the localized seizures was significantly smaller for ESI + connectivity than for ESI power which points out that FC enhances the robustness against intra-patient variability of ictal imaging. The measure was also lower than compared to a previous study that assessed spatial dispersion (Pellegrino et al., 2016), both for ESI + connectivity and ESI power.

Although spatial dispersion is a good measure for intra-patient robustness, the example of PAT 8 showed that the useful information obtained by the spatial dispersion could be limited due to possible outliers in the data. Using this technique prospectively, we would not be able to discriminate true outliers from seizures that could possibly have originated elsewhere. Therefore, careful interpretation of the individual seizure results is required.

### 4.3 Subgroup analysis based on resected volume

Although we found that the ESI + connectivity method scored significantly better for large than for small resections, the median distance for small resections is, like for large resections, 0 mm, indicating that the method also performs well for small resections. Both the fact that no significant difference between the percentages of correctly localized seizures per patient was found and the fact that the distance to the center of the RZ was smaller for small resections, confirms that there is no actual difference in performance for small and large resections.

### 4.4 Considerations and limitations

A method for SOZ localization should preferably be non-invasive, objective, fast, and accurate. First, since only scalp EEG and an MR image of the patient's head (to generate the individual head model) are needed, this method is non-invasive. Second, the pipeline is not completely objective yet, since the initial epoch and FOI selection require interaction with a human expert. It should be further investigated how this influences the results and how this can be made user-independent (see also Supplementary material A.3). After this initial input, though, there are no subjective parameters left. Third, we did not report results on the speed of the algorithm. However, it would be possible to build a completely automated pipeline, running calculations in the background (taking approximately 1 h for a new patient and less than a minute for a new seizure of this patient). This way, only a few minutes of the epileptologist's time are needed for initial epoch and FOI selection. Lastly, we have found that the accuracy of ESI + connectivity is high. All these factors point out that the method could be a useful aid in the presurgical evaluation of epilepsy.

There are also some limitations to our study. We compared the selected SOZ and the resected area in the brain of the patient to test whether the method worked correctly. However, using the RZ as gold standard provides a suboptimal validation. First, it is often an overestimation of the real ground truth. If a patient is seizure-free, we can assume that the SOZ was somewhere inside the RZ, but we do not know exactly where. Second, the patient being seizure-free does not prove that a specific analyzed seizure truly originated in the RZ. Finally, error estimation using Euclidean distance does not take brain anatomy into account. It could be that e.g. for a small LE larger than 0 mm, an important fissure is crossed, possibly making the estimation uninformative. Therefore, LE, defined as the Euclidean distance to the border of the RZ, is not a perfect measure for validation. Fortunately, most of the SOZ estimations in this study (for ESI + connectivity) were inside the RZ, making this limitation less of a problem. In future research, validation results could be correlated to brain anatomy and to findings of intracranial EEG (iEEG) recordings, which can provide a more precise truth. It needs to be evaluated whether both methods point to the same brain region, provided that the (iEEG) samples the SOZ.

In this study, we only included patients that had Engel Class I outcome at least 1 year after surgery. This allowed for assessing the percentage of SOZs that are estimated inside or close to the RZ in seizure-free patients. However, we did not estimate the SOZ and compare this to the RZ in patients that were not rendered seizure-free (Engel Classes II–IV). It is important, and part of future research, to investigate whether in these patients a SOZ differing from the RZ is found or not.

Although most included patients suffered from temporal lobe epilepsy, there were 3 patients with extratemporal epilepsy (PAT1, PAT8, PAT20). Also in these patients, the presented method performed generally well (LE ≤ 10 mm in 7/7 seizures for PAT 1; LE = 0 mm in 4/6 seizures for PAT 8 and LE = 0 mm in 12/12 seizures for PAT 20). Albeit not perfect, this indicates that the application domain of the approach lies beyond temporal lobe epilepsy. Actually, we did not find any apparent relationship between performance of the ESI + connectivity algorithm and patient characteristics, ictal patterns or EEG quality. More validation in a larger, more heterogeneous population group is required to confirm this, but this finding already points out the possibly wide application area of the method.

Other methodological considerations concerning the MVAR model order, the inverse solution technique and the spatial extent of the SOZ can be found in the Supplementary material A.3.

## 5 Conclusion

We showed that it is possible to estimate the SOZ from clinical or low-density scalp EEG with high accuracy using ESI and subsequent functional connectivity analysis. Moreover, the proposed method is non-invasive and requires, after initial epoch and frequency selection, minimal user-dependent input. Altogether, the method could serve as a useful tool for SOZ localization in the presurgical evaluation of epilepsy. Larger studies are warranted, notably with more extratemporal epilepsies and localization correlation with a range of different outcomes.

## Appendix A Supplementary material

Supplementary material. Image 1
