# Supplementary material for: EEG source connectivity to localize the seizure onset zone in patients with drug resistant epilepsy
Source: Neuroimage Clin. 2017 Sep 14;16:689–98. doi: 10.1016/j.nicl.2017.09.011 (PMC5633847; doi:10.1016/j.nicl.2017.09.011)
Supplement: Supplementary file 1 — Supplementary material. [file mmc1.docx]

# Appendix

## Analyzed seizures per patient and epoch selection

Table A.1: Overview of the analyzed seizures for every patient. The selected epoch w.r.t. to the marked seizure onset (time of interest, TOI), the seizure frequency and possible extra preprocessing steps are mentioned. ICA was applied on clear eyeblink artifacts, unless mentioned otherwise. Interp = interpolation, HB = heartbeat.

| **PAT** |  | **Seizure** | | | | | | | | | | | |
| --- | --- | --- | --- | --- | --- | --- | --- | --- | --- | --- | --- | --- | --- |
|  |  | **1** | **2** | **3** | **4** | **5** | **6** | **7** | **8** | **9** | **10** | **11** | **12** |
| **1** | **TOI (s)** | [1 5] | [-1 3] | [8.8 10] | [3 8] | [1.5 4] | [5 10] | [0 4] |  |  |  |  |  |
|  | **FOI (Hz)** | [4 7] | [4 7] | [4 7] | [1 7] | [4 12] | [4 7] | [4 12] |  |  |  |  |  |
|  | **extra**  **preproc.** | - | - | filter  1-10 Hz | Interp  T9 | Interp  T9,FT10 | filter  1-10 Hz | Interp  Fpz |  |  |  |  |  |
| **2** | **TOI (s)** | [-2 1.5] | [0 3] | [1 6] | [-0.5 4.5] | [0 3.5] | [0 2] | [0 5] |  |  |  |  |  |
|  | **FOI (Hz)** | [12 15] | [1 8] | [1 8] | [2 6] | [2 8] | [2 6] | [1 8] |  |  |  |  |  |
|  | **extra**  **preproc.** | Interp  T5 | - | ICA | - | - | - | - |  |  |  |  |  |
| **3** | **TOI (s)** | [-2 3] | [-2 1]] | [3.5 8] | [0 5] |  |  |  |  |  |  |  |  |
|  | **FOI (Hz)** | [4 10] | [4 7] | [4 7] | [4 7] |  |  |  |  |  |  |  |  |
|  | **extra**  **preproc.** | filter  1-10 Hz | ICA | Interp  FT10,C3 | - |  |  |  |  |  |  |  |  |
| **4** | **TOI (s)** | [0 4] | [3 6] | [8 10] | [6 11] | [11 15] |  |  |  |  |  |  |  |
|  | **FOI (Hz)** | [4 7] | [4 7] | [4 7] | [4 7] | [4 7] |  |  |  |  |  |  |  |
|  | **extra**  **preproc.** | - | Interp  C3 | Interp  C5,O1,  Oz | Interp  T5,O1;  ICA | - |  |  |  |  |  |  |  |
| **5** | **TOI (s)** | [2.8 4.8] | [2 7] | [5 10] | [1 6] |  |  |  |  |  |  |  |  |
|  | **FOI (Hz)** | [4 8] | [4 8] | [4 8] | [4 9] |  |  |  |  |  |  |  |  |
|  | **extra**  **preproc.** | Interp  P4,T3 | Interp  T6 | - | - |  |  |  |  |  |  |  |  |
| **6** | **TOI (s)** | [-5 0] | [-5 0] | [-4 -2] | [-1.8 0.5] | [6 10] | [0 5] | [1 3.5] |  |  |  |  |  |
|  | **FOI (Hz)** | [8 15] | [8 15] | [8 15] | [8 15] | [4 7] | [3 10] | [3 10] |  |  |  |  |  |
|  | **extra**  **preproc.** | ICA | - | ICA | Interp  FT10 | filter  1-10 Hz | filter  1-10 Hz | filter  1-10 Hz |  |  |  |  |  |
| **7** | **TOI (s)** | [-1 1] | [11.1 12.8] | [18 20] |  |  |  |  |  |  |  |  |  |
|  | **FOI (Hz)** | [1 4] | [1 4] | [2 7] |  |  |  |  |  |  |  |  |  |
|  | **extra**  **preproc.** | filter  1-10 Hz | filter  1-10 Hz | - |  |  |  |  |  |  |  |  |  |
| **8** | **TOI (s)** | [0 3] | [-3 2] | [-3 2] | [1.7 6] | [0 3] | [0 4] |  |  |  |  |  |  |
|  | **FOI (Hz)** | [8 15] | [8 15] | [8 15] | [8 15] | [8 10] | [8 10] |  |  |  |  |  |  |
|  | **extra**  **preproc.** | ICA | - | - | - | filter  1-10 Hz | filter  1-10 Hz |  |  |  |  |  |  |
| **9** | **TOI (s)** | [-1 1.9] |  |  |  |  |  |  |  |  |  |  |  |
|  | **FOI (Hz)** | [1 3] |  |  |  |  |  |  |  |  |  |  |  |
|  | **extra**  **preproc.** | Interp Oz, ICA |  |  |  |  |  |  |  |  |  |  |  |
| **10** | **TOI (s)** | [-5 0] | [15 20] | [0 5] | [4 9] |  |  |  |  |  |  |  |  |
|  | **FOI (Hz)** | [1 7] | [1 7] | [10 15] | [1 7] |  |  |  |  |  |  |  |  |
|  | **extra**  **preproc.** | - | - | - | filter  1-10 Hz |  |  |  |  |  |  |  |  |
| **11** | **TOI (s)** | [5 10] | [2.5 7.5] | [2.2 7.2] | [4 6] | [4 7] | [2 5] |  |  |  |  |  |  |
|  | **FOI (Hz)** | [4 7] | [4 7] | [1 7] | [4 7] | [1 7] | [1 5] |  |  |  |  |  |  |
|  | **extra preproc.** | Interp T10, ICA and filter  1-10 Hz | Interp T10, FT10;  ICA;filter  1-10 Hz | Interp T10, ICA and filter  1-10 Hz | ICA | ICA | ICA |  |  |  |  |  |  |
| **12** | **TOI (s)** | [0 3] | [-1 3] | [0 3] | [0 2.5] | [0 2.5] | [-1.5 0] | [0 5] | [-1 1] |  |  |  |  |
|  | **FOI (Hz)** | [4 7] | [4 7] | [3 6] | [3 6] | [4 7] | [3 6] | [4 7] | [3 6] |  |  |  |  |
|  | **extra**  **preproc.** | Interp  T10;  ICA | ICA | - | - | - | - | ICA | Interp O1 |  |  |  |  |
| **13** | **TOI (s)** | [0 2] |  |  |  |  |  |  |  |  |  |  |  |
|  | **FOI (Hz)** | [10 15] |  |  |  |  |  |  |  |  |  |  |  |
|  | **extra**  **preproc.** | - |  |  |  |  |  |  |  |  |  |  |  |
| **14** | **TOI (s)** | [1 2.5] | [4 7] | [9.5 14.5] | [0.2 3.8] |  |  |  |  |  |  |  |  |
|  | **FOI (Hz)** | [1 4] | [3 7] | [3 7] | [3 7] |  |  |  |  |  |  |  |  |
|  | **extra**  **preproc.** | ICA | Interp O1, T9 | Interp O1 | Interp O1, filter  1-10 Hz |  |  |  |  |  |  |  |  |
| **15** | **TOI (s)** | [0 5] | [5 10] | [0.5 2.5] |  |  |  |  |  |  |  |  |  |
|  | **FOI (Hz)** | [1 5] | [1 5] | [1 5] |  |  |  |  |  |  |  |  |  |
|  | **extra**  **preproc.** | ICA | ICA | ICA |  |  |  |  |  |  |  |  |  |
| **16** | **TOI (s)** | [0 3.5] | [-1.5 3.5] | [0 5] | [0 5] |  |  |  |  |  |  |  |  |
|  | **FOI (Hz)** | [4 7] | [4 7] | [4 7] | [4 7] |  |  |  |  |  |  |  |  |
|  | **extra**  **preproc.** | - | ICA | - | Interp Oz |  |  |  |  |  |  |  |  |
| **17** | **TOI (s)** | [17.5 20] | [0 3] | [4 7] |  |  |  |  |  |  |  |  |  |
|  | **FOI (Hz)** | [1 4] | [1 4] | [1 4] |  |  |  |  |  |  |  |  |  |
|  | **extra**  **preproc.** | filter  1-10 Hz | ICA | filter  1-10 Hz |  |  |  |  |  |  |  |  |  |
| **18** | **TOI (s)** | [3 7] | [0 5] | [0 5] |  |  |  |  |  |  |  |  |  |
|  | **FOI (Hz)** | [1 4] | [1 4] | [1 4] |  |  |  |  |  |  |  |  |  |
|  | **extra**  **preproc.** | - | - | - |  |  |  |  |  |  |  |  |  |
| **19** | **TOI (s)** | [2 6] |  |  |  |  |  |  |  |  |  |  |  |
|  | **FOI (Hz)** | [1 5] |  |  |  |  |  |  |  |  |  |  |  |
|  | **extra**  **preproc.** | filter  1-10 Hz |  |  |  |  |  |  |  |  |  |  |  |
| **20** | **TOI (s)** | [-3 0] | [-5 0] | [4 7.5] | [0 5] | [0 4] | [0 4] | [0 2] | [3.5 8.5] | [0 5] | [-1 4] | [0 5] | [0 5] |
|  | **FOI (Hz)** | [1 5] | [25 30] | [20 30] | [20 30] | [7 15] | [15 25] | [15 25] | [20 25] | [7 15] | [7 10] | [15 25] | [7 15] |
|  | **extra**  **preproc.** | - | - | - | - | - | ICA | ICA | - | Interp  Fp2, Tp10 | - | Interp  Fp1 | - |
| **21** | **TOI (s)** | [5 9.5] | [4 9] |  |  |  |  |  |  |  |  |  |  |
|  | **FOI (Hz)** | [1 5] | [1 5] |  |  |  |  |  |  |  |  |  |  |
|  | **extra**  **preproc.** | filter  1-10 Hz | filter  1-10 Hz |  |  |  |  |  |  |  |  |  |  |
| **22** | **TOI (s)** | [4.5 9.5] | [0 5] | [0 4] | [-1 1.5] | [0 5] | [-0.5 4.5] | [3.8 8.7] | [1.8 6] | [0 5] |  |  |  |
|  | **FOI (Hz)** | [1 7] | [1 7] | [1 7] | [7 12] | [1 12] | [1 12] | [1 15] | [1 12] | [1 7] |  |  |  |
|  | **extra**  **preproc.** | - | - | - | - | - | - | - | - | - |  |  |  |
| **23** | **TOI (s)** | [0 5] | [0 2] | [0 5] |  |  |  |  |  |  |  |  |  |
|  | **FOI (Hz)** | [1 10] | [4 7] | [3 8] |  |  |  |  |  |  |  |  |  |
|  | **extra**  **preproc.** | - | filter  1-10 Hz | ICA |  |  |  |  |  |  |  |  |  |
| **24** | **TOI (s)** | [0 3.5] |  |  |  |  |  |  |  |  |  |  |  |
|  | **FOI (Hz)** | [4 7] |  |  |  |  |  |  |  |  |  |  |  |
|  | **extra**  **preproc.** | ICA |  |  |  |  |  |  |  |  |  |  |  |
| **25** | **TOI (s)** | [0 5] |  |  |  |  |  |  |  |  |  |  |  |
|  | **FOI (Hz)** | [4 12] |  |  |  |  |  |  |  |  |  |  |  |
|  | **extra preproc.** | - |  |  |  |  |  |  |  |  |  |  |  |
| **26** | **TOI (s)** | [0 5] |  |  |  |  |  |  |  |  |  |  |  |
|  | **FOI (Hz)** | [1 7] |  |  |  |  |  |  |  |  |  |  |  |
|  | **extra**  **preproc.** | - |  |  |  |  |  |  |  |  |  |  |  |
| **27** | **TOI (s)** | [1 5] |  |  |  |  |  |  |  |  |  |  |  |
|  | **FOI (Hz)** | [24 26] |  |  |  |  |  |  |  |  |  |  |  |
|  | **extra**  **preproc.** | ICA (HB) |  |  |  |  |  |  |  |  |  |  |  |

## A.2 Spatial spread figures of all patients

See figures.


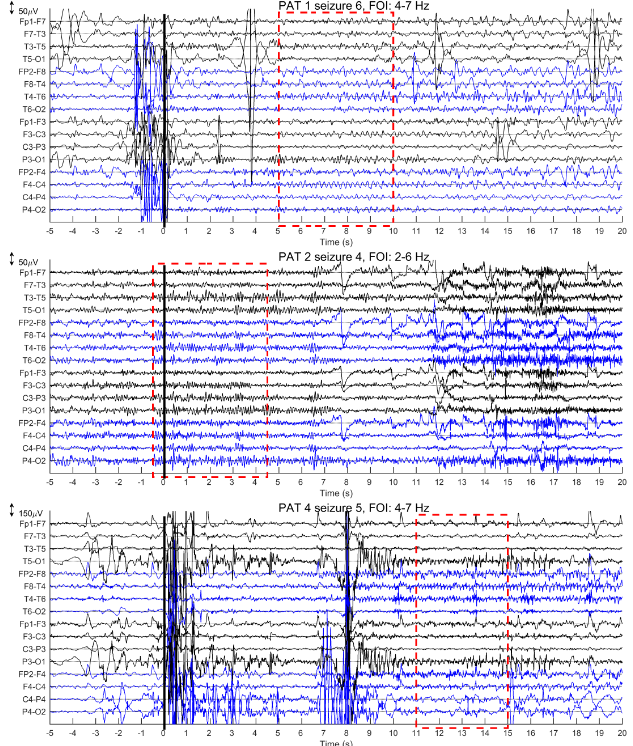


Figure A.1


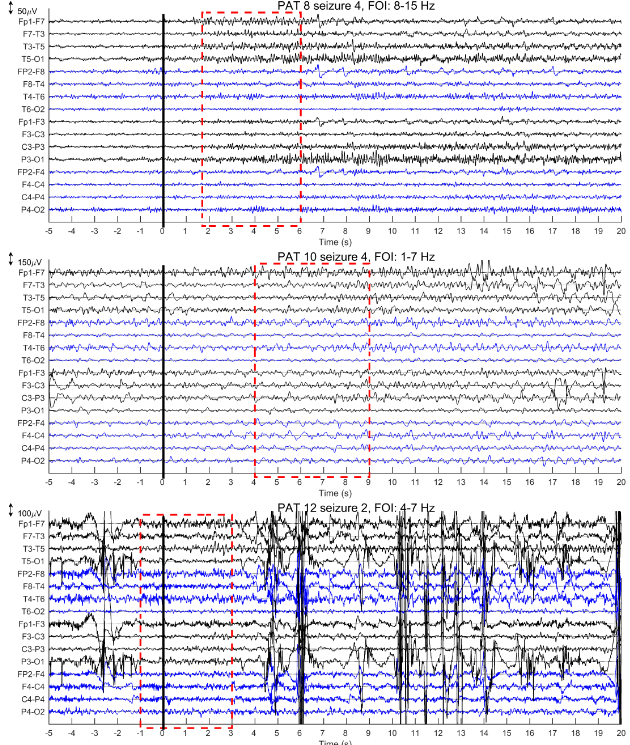


Figure A.2


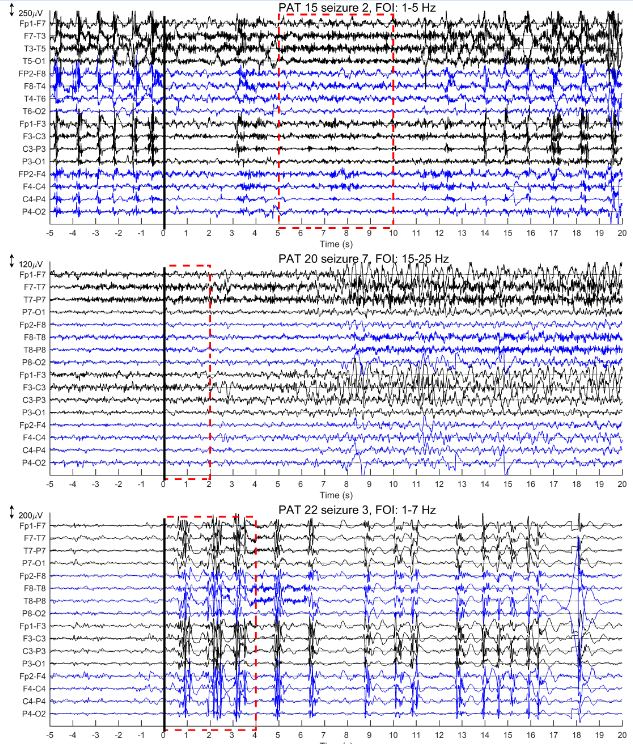


Figure A.3


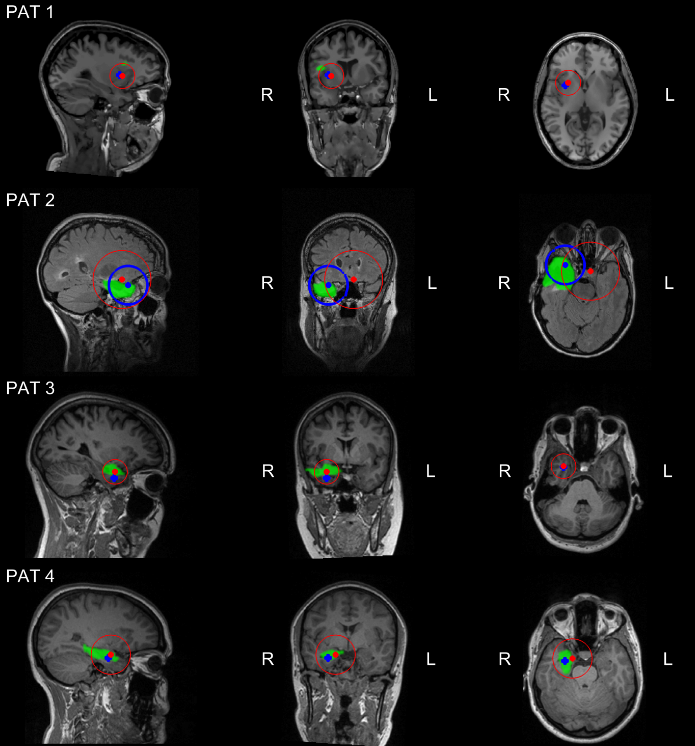


Figure A.4


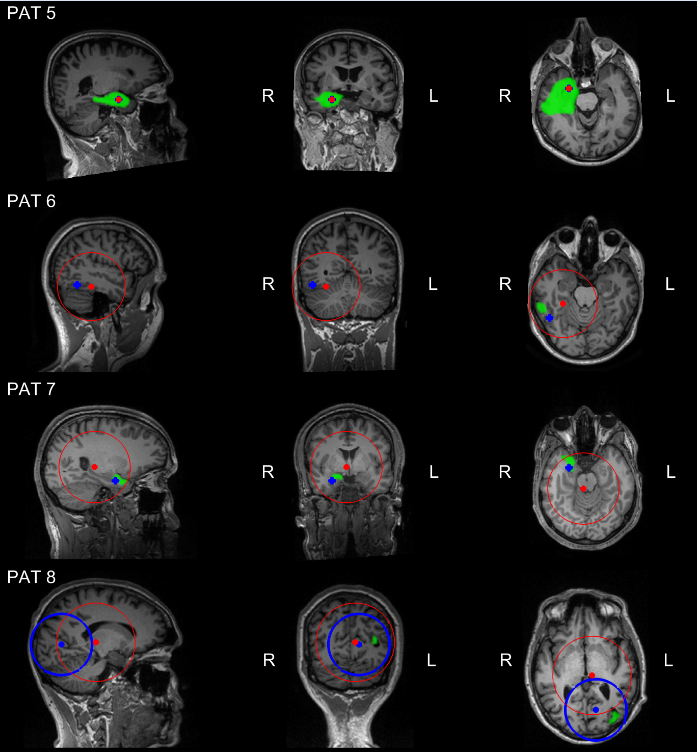


Figure A.5


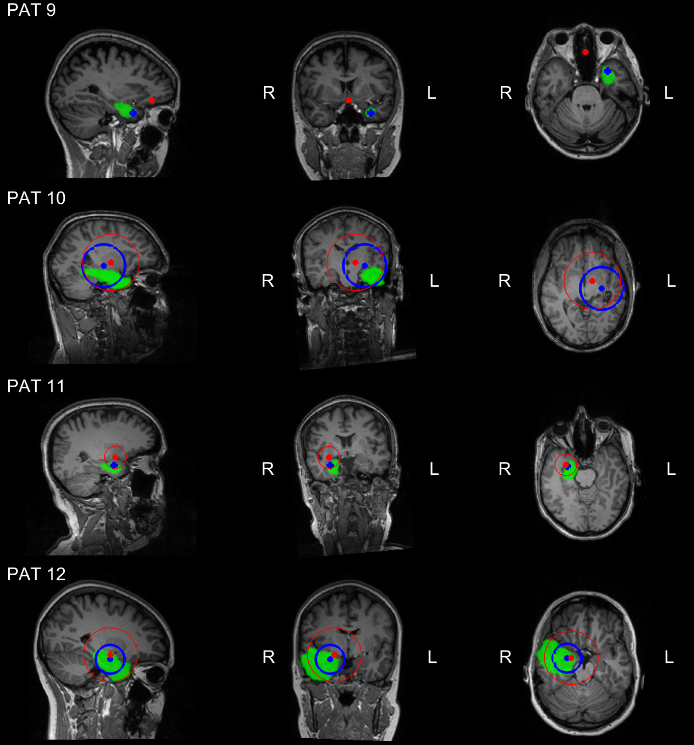


Figure A.6


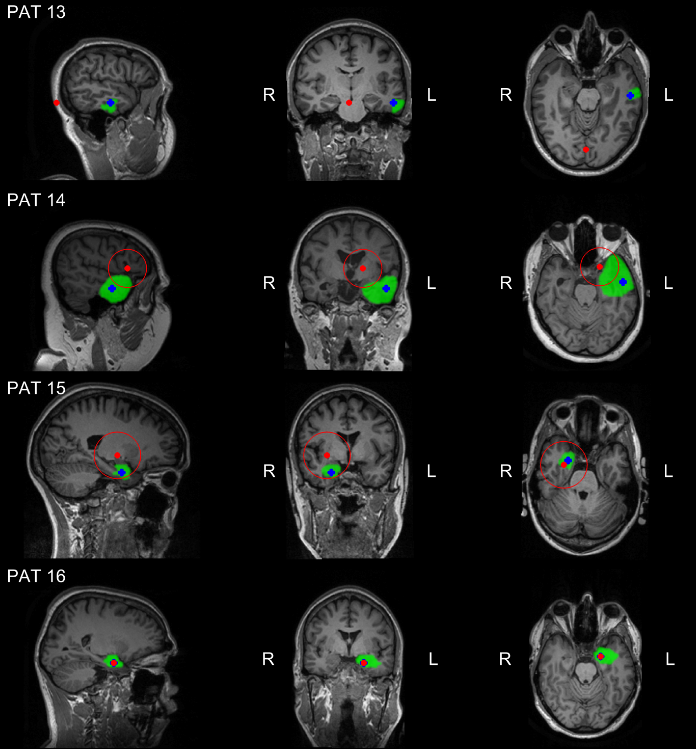


Figure A.7


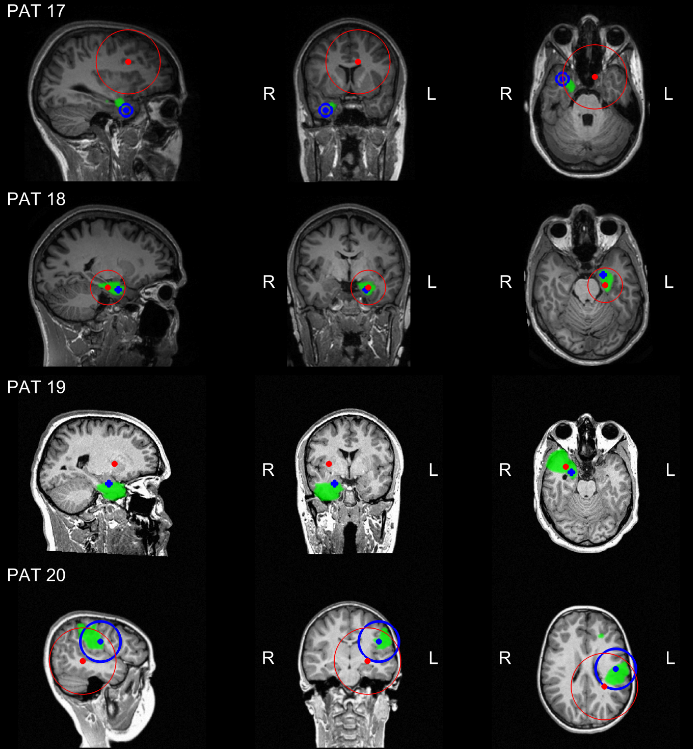


Figure A.8


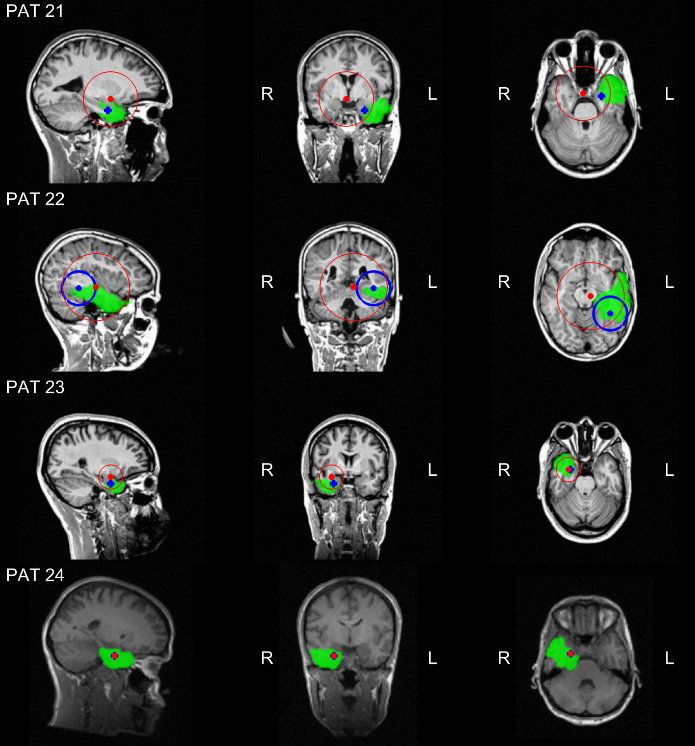


Figure A.9


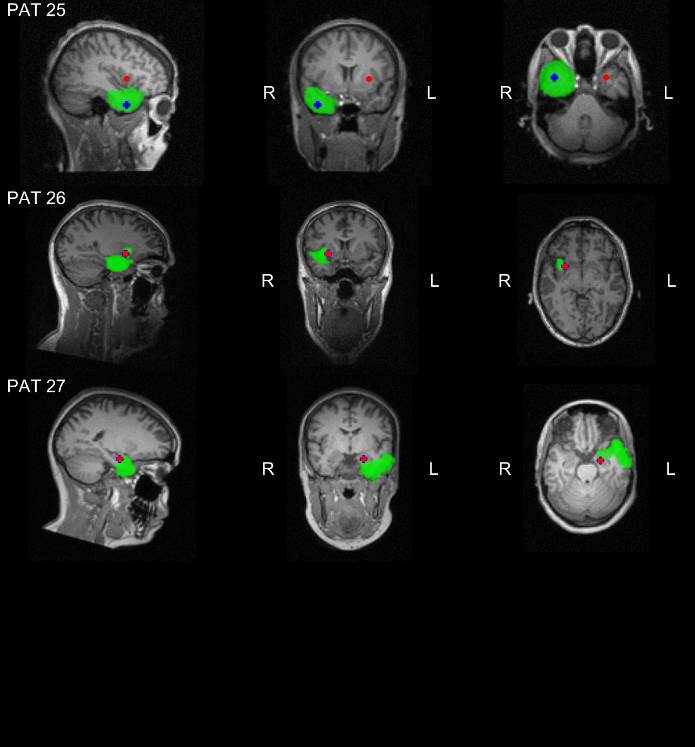


Figure A.10

## A.3 Extra methodological considerations

Since interaction with a human expert is needed to select the ictal epochs, there is some subjectivity left in the methods and it remains to be investigated how this influences the results and how robust the method is for changes in epoch/FOI selection. In most patients with clear data and clear constant ictal discharges, we found that epoch selection is not critical and results are consistent over time and selected fragment. Yet, it is harder to compare with artifactual seizures or seizures that have a changing pattern over time. A rigorous validation is needed for this epoch selection. One possibility could be to perform an automated deterministic epoch selection, based on the temporal and spectral behavior of the EEG during the seizure. Such an automatic selection is not trivial because often it is hard to discriminate between artifact and ictal activity, because they can have similar morphology and can have an overlapping spectral content. Nevertheless, the goal of this study was to show that it is possible to localize or indicate the SOZ based on low-density ictal scalp EEG data consistently over many seizures, rather than to build a completely automated pipeline.

It could be argued that the model order of the TVAR model could be determined based on e.g. the Akaike Information Criterion (AIC) or Bayesian Information Criterion. For some patients, we did calculate the optimal model order with the AIC for several seizures, and generally found a value between 4 and 10. Since the computation time substantially increased to calculate the optimal model order, we opted to fix the model order to 10 taking into account the fact that a model order that is slightly too low (connections cannot be found) is worse than a model order that is slightly too high (usually insignificant) (Schlindwein and Evans, 1990).

The spatial sampling of the grid in source space was 4 mm and the inverse solution was calculated with LORETA at each of these gridpoints. However, since LORETA offers a smooth solution, the true spatial resolution is worse than 4 mm. Yet, we need sufficient spatial sampling in order to make sure that the true SOZ is sampled. Other inverse techniques, such as beamformers, could provide more focal solutions (Russell and Koles, 2007), and thus with a higher spatial resolution. It would be interesting for future research to investigate the influence of different inverse solution techniques on the performance of ESI + connectivity.

The SOZ localized by our method consists of one grid point. This way, we give an indication of the location of the true SOZ, but not of its spatial extent. An interesting extension of the current method would be to also provide a measure for the spatial extent of the SOZ, which might be based on the power spectrum of the neighboring sources.

## References

Russell J.P. and Koles Z.J., A comparison of LORETA and the Borgiotti-Kaplan Beamformer in simulated EEG source localization with a realistic head model, International Journal of Bioelectromagnetism 9, 2007, 112–113.

Schlindwein F.S. and Evans D.H., Selection of the order of autoregressive models for spectral analysis of doppler ultrasound signals, *Ultrasound Med. Biol.* **16**, 1990, 81–91, <https://doi.org/10.1016/0301-5629(90)90089-U>.

## Figure legends

Figure A. 1: Examples of epoch selection. A bipolar montage of the preprocessed EEG from 5s before until 20s after seizure onset is shown. The red rectangle indicates the chosen time frame. The used frequency band of interest for analysis is shown in the title. Note that the actual segment selection also happened based on channels that are not shown in this montage (e.g. the midline electrodes).

Figure A. 2: Examples of epoch selection. A bipolar montage of the EEG from 5s before until 20s after seizure onset is shown. The red rectangle indicates the chosen time frame. The used frequency band of interest for analysis is shown in the title. Note that the actual segment selection also happened based on channels that are not shown in this montage (e.g. the midline electrodes).

Figure A. 3: Examples of epoch selection. A bipolar montage of the EEG from 5s before until 20s after seizure onset is shown. The red rectangle indicates the chosen time frame. The used frequency band of interest for analysis is shown in the title. Note that the actual segment selection also happened based on channels that are not shown in this montage (e.g. the midline electrodes).

Figure A.4-A10: Spatial spread of all patients. The standard distance of patients that had only one analyzed seizure is shown as zero (no circle). Remark that the slices shown in the visualization are optimized for the ESI+connectivity results. As a consequence, some ESI power results may seem to lie outside source space, but this is not the case.
